# "In vitro" activities of antimycobacterial agents against *Mycobacterium avium *subsp. *paratuberculosis *linked to Crohn's Disease and Paratuberculosis

**DOI:** 10.1186/1476-0711-5-27

**Published:** 2006-11-15

**Authors:** Stefania Zanetti, Paola Molicotti, Sara Cannas, Silvia Ortu, Niyaz Ahmed, Leonardo A Sechi

**Affiliations:** 1 Dipartimento di Scienze Biomediche, Sezione di Microbiologia Sperimentale e Clinica, Università degli studi di Sassari, Viale S. Pietro 43/B, 07100 Sassari, Italy; 2 ISOGEM Collaborative Network on Genetics of Mycobacteria (The International Society for Genomic and Evolutionary Microbiology, Sassari, Italy); 3 Pathogen Evolution Group, Center for DNA Fingerprinting and Diagnostics, Hyderabad, India

## Background

Crohn's disease, a human disease similar to paratuberculosis in animals is the most painful and devastating disease that may involve infection with *M. avium *subsp. *paratuberculosis *(MAP), different genetic polymorphisms and an immune dysregulation syndrome [1-5]. Treatment of Crohn's disease is most commonly based on 5-aminosalicylic acid (5-ASA) compounds, corticosteroids, and immunosuppressive agents. Recently, biological therapies using monoclonal antibodies against inflammatory cytokines have shown some positive results [6]. However, all these therapies treat the symptoms not the cause of the disease [6].

Antimycobacterial therapy against MAP has recently showed promising results [7]. In fact, several authors provided convincing evidence that antimycobacterial compounds may influence the course of the disease [4].

Currently, there are no antibiotics approved for the treatment of Johne's disease or Crohn's disease. Considering the worldwide magnitude and impact of health concerns caused by MAP, we studied antimycobacterial activity of different antituberculosis agents *in vitro *against MAP bacilli of human and veterinary origins. Moreover, we were interested to investigate the variability of susceptibility to antimycobacterial compounds of the MAP isolates with respect to growth in agar medium and Congo red (a planar hydrophobic molecule that binds to lipids and lipoproteins, which are abundant in mycobacterial cell walls) staining properties.

All the strains we used in this study were isolated from humans and animals, the ATCC strain was a gift of Peter Overduin, RIVM, Bilthoven, The Netherlands. Conventional methods and a IS900 PCR test and sequencing of the amplification products reconfirmed specific identity of all the strains [1]. The strains (7 humans and 5 animals) were tested against antimicrobial agents of several classes (streptomycin, ciprofloxacin, ethambutol, dapsone, rifampin, rifabutin and clarithromycin) which are commonly used for the treatment of mycobacterial diseases. Bacteria were cultivated in Middlebrook7H9 medium supplemented with Middlebrook ADC enrichment and Mycobactin J following conventional methods [1,8]. MIGIT bottles were inoculated with 10^4 ^to 10^5 ^CFU/ml. The antimycobacterial activity was determined by the BACTEC MGIT 960 method with a modified protocol [8] by adding mycobactin J and reading the mycobacterial growth up to 90 days.

The concentration of the chemical agents tested were as follows: streptomycin 0,5, 1, 2 and 4 μg/ml, ciprofloxacin 0,5, 1, 2, 4, 8 and 16 μg/ml, ethambutol 1, 2, 4, 8 and 16 μg/ml, rifampin 0.5, 1 and 2 μg/ml, rifabutin 0.5, 1, 2 μg/ml, clarithromycin 2, 4, 8, 16 and 32 μg/ml and dapsone 8, 16 and 32 μg/ml. Experiments were repeated three times. The MICs were interpreted by measuring the change in the daily GI (DGI) for the drug-containing vials compared with the GI for the control vials,

Activities of the antibiotics tested in this work correlated well with those described in previous reports on susceptibility of mycobacteria to antimicrobial agents except for clarythromicin [8,9]. Strains were prepared for the agar assay and plated in triplicate on 7H10 agar medium containing 100 μg/ml of Congo Red (Sigma Chemical Co. St. Louis, Mo, USA). Plates were incubated for 40–60 days to observe colony morphology: rough or smooth, opaque or transparent, red or white [11].

We observed that streptomycin, rifabutin and rifampin, revealed a characteristic low MIC of 0,5 to 4 μg/ml, also ciprofloxacin shoed a low MIC (range 0,5–16) whereas, dapsone's MIC was very high at 32 μg/ml. MICs of two other drugs were 2–16 μg/ml and 2–32 μg/ml for ethambutol and clarithtromycin respectively. Susceptibility data on human and animal isolates were carefully compared and summarized in Table 1. Briefly, for streptomycin the MIC range was of 0,5-2 μg/ml in both animal and human strains, for ciprofloxacin it was at 0.5–16 μg/ml, independently of the source of isolation, while rifampin produced a MIC of 0.5–1 μg/ml in human and bovine strains and of >2 μg/ml for 2 bovine and 1 animal isolate. Rifabutin MIC was very similar to rifampin. Also ethambutol generated a MIC of 2–8 μg/ml in human strains and 4–16 μg/ml in animal strains. The highest MIC values were observed for dapsone: 32 μg/ml for human strains but 16 μg/ml for animal isolates. Clarithromycin showed a MIC ranging from 2 to 32 μg/ml independently of the isolation source, which is in contrast with previous reports [9-11] where they report a lower MIC of 0.5–4 μg/ml, probably due to differences in the pH medium used. Our results concerning growth in Congo red medium agar showed prevalently a rough transparent colony morphotype with white coloration, whereas no smooth opaque colonies with red coloration on Congo Red agar were detected as previously observed except that for ATCC 43015 [11], moreover, no correlation with antimicrobial susceptibilities was observed.

**Table 1:** *In vitro *activity of several drugs against MAP strains

**Drugs **(μg/ml)							
**Strains**	**STR**	**CPX**	**EMB**	**DPS**	**RIF**	**RIB**	**CLA**

**ATCC 43015**	0.5	0.5	2	16	0.5	2	2
**182 human**	2	4	4	32	0.5	1	4
**516 human**	2	8	4	16	1	1	16
**517 human**	2	4	8	32	>2	>2	16
**090 human**	2	8	8	16	1	1	16
**186 human**	1	4	2	32	1	1	4
**064 human**	1	2	4	32	0.5	1	4
**387 cow**	2	2	4	16	0.5	0.5	2
**436 cow**	2	4	8	16	>2	>2	16
**088 cow**	1	16	16	16	>2	>2	32
**033 cow**	2	4	16	16	0.5	0,5	16
**518 cow**	2	8	16	16	1	2	2

**STR**: Streptomycin, **CPX**: Ciprofloxacin, **EMB**: Ethambutol, **DPS**: Dapsone, **RIF**: Rifampin, **RIB**: Rifabutin, **CLA**: Clarithromycin. Source of isolation of strains is also indicated. Numbers represent the MIC value for each drug.

A number of antimicrobial agents have been used to combat flare ups in Crohn's Disease. These have included Ciprofloxacin and metronidazole, used either alone or in combination. In recent studies, Crohn's patients were treated with 3 antibiotics for a minimum of 24 months [4,7].

MAP multiplies much more slowly than *M. tuberculosis*, and so its treatment length should probably be double if not triple of that of the later. Other mycobacterial diseases, such as leprosy, also require prolonged treatment. Infection of the lung due to *Mycobacterium avium complex *and other mycobacteria, in patients who are not immunocompromised, has been treated for about 15 months to five years [12]. Moreover, considering that clarithromycin and ciprofloxacin are widely used antibiotics, strains resistant to these drugs can be very common. Up to now, there is a urgent need of new information on the activity of available antibiotic regimens against MAP.

In conclusion, these results might represent a good starting point to consider different drugs for treatment of the disease forms where involvement of MAP is suspected, in particular Crohn's disease in humans.

## References

[B1] Sechi LA, Scanu AM, Molicotti P, Cannas S, Mura M, Dettori G, Fadda G, Zanetti S (2005). Detection and isolation of *Mycobacterium avium *subspecies *paratuberculosis *from intestinal mucosal biopsies of patients with and without Crohn's disease in Sardinia. Am J Gastroenterol.

[B2] Bull TJ, McMinn EJ, Sidi-Boumedine K, Skull A, Durkin D, Neild P, Rhodes G, Pickup R, Hermon Taylor J (2003). Detection and verification of *Mycobacterium avium *subsp. *paratuberculosis *in fresh ileocolonic mucosal biopsy specimens from individuals with and without Crohn's disease. J Clin Microbiol.

[B3] Naser SA, Ghobrial G, Romero C, Valentine JF (2004). Culture of *Mycobacterium avium *subspecies *paratuberculosis *from the blood of patients with Crohn's disease. Lancet.

[B4] Hermon-Taylor J (2002). Treatment with drugs active against *Mycobacterium avium *subspecies *paratuberculosis *can heal Crohn's disease: more evidence for a neglected public health tragedy. Dig Liver Dis.

[B5] Greenstein RJ, Collins MT (2004). Emerging pathogens: is *Mycobacterium avium *subspecies *paratuberculosis *zoonotic?. Lancet.

[B6] Blonski W, Lichtenstein GR (2006). Complications of biological therapy for inflammatory bowel diseases. Curr Opin Gastroenterol.

[B7] Borody TJ, Leis S, Warren EF, Surace R (2002). Treatment of severe Crohn's disease using antimycobacterial triple therapy-approaching a cure?. Dig Liver Dis.

[B8] Ardito F, Posteraro B, Sanguinetti M, Zanetti S, Fadda G (2001). Evaluation of BACTEC Mycobacteria Growth Indicator Tube (MGIT 960) automated system for drug susceptibility testing of *Mycobacterium tuberculosis*. J Clin Microbiol.

[B9] Rastogi N, Goh KS, Labrousse V (1992). Activity of clarithromycin compared with those of other drugs against *Mycobacterium paratuberculosis *and further enhancement of its extracellular and intracellular activities by ethambutol. Antimicrob Agents Chemother.

[B10] Rastogi N, Goh KS, Berchel M, Bryskier A (2000). In vitro activities of the ketolides telithromycin (HMR 3647) and HMR 3004 compared to those of clarithromycin against slowly growing mycobacteria at pHs 6.8 and 7.4. Antimicrob Agents Chemother.

[B11] Parrish NM, Ko CG, Dick JD, Jones PBm, Ellingson JL (2004). Growth, Congo Red agar colony morphotypes and antibiotic susceptibility testing of *Mycobacterium avium *subspecies *paratuberculosis*. Clin Med Res.

[B12] Koh WJ, Kwon OJ, Lee KS (2005). Diagnosis and treatment of nontuberculous mycobacterial pulmonary diseases: a Korean perspective. J Korean Med Sci.

